# Neonatal *Staphylococcus Aureus* Sepsis: a 20-year Western Australian experience

**DOI:** 10.1038/s41372-022-01440-3

**Published:** 2022-06-25

**Authors:** Rachel Shadbolt, Michael Lee Shee We, Rolland Kohan, Michelle Porter, Gayatri Athalye-Jape, Elizabeth Nathan, Damber Shrestha, Tobias Strunk

**Affiliations:** 1 Medical School, University of Western Australia, Perth, WA Australia; 2 Centre for Neonatal Research and Education and Division of Paediatrics, Medical School, University of Western Australia, Perth, WA Australia; 3 Telethon Kids Institute, Perth, WA Australia; 4 Neonatal Directorate, Child and Adolescent Health Service, Perth, WA Australia; 5 Microbiology Department, PathWest Laboratories, Perth, WA Australia; 6 Division of Obstetrics and Gynaecology, University of Western Australia and the Women and Infants Research Foundation, King Edward Memorial Hospital, Perth, WA Australia

**Keywords:** Paediatrics, Bacterial infection, Risk factors

## Abstract

**Objectives:**

The purpose of this study was to characterise neonatal *Staphylococcus aureus* (SA) sepsis in Western Australia (WA) between 2001 and 2020 at the sole tertiary neonatal intensive care unit (NICU), examine risk factors for sepsis in the cohort, and compare short- and long-term outcomes to control infants without any sepsis.

**Methods:**

Retrospective cohort study at the Neonatal Directorate at King Edward Memorial Hospital (KEMH) and Perth Children’s Hospital, using electronic databases and patient medical records.

**Results:**

The overall incidence of SA sepsis was 0.10 per 1000 live births (62/614207). From 2001 to 2010 the incidence was 0.13/1000 live births, reducing to 0.07/1000 live births from 2011 to 2020. SA was most frequently isolated from endotracheal aspirates, and infants with SA sepsis had longer median duration of ventilatory support than those without any sepsis (31 days vs 18 days respectively, *p* < 0.001). In our cohort, SA sepsis was associated with worse neurodevelopmental outcomes compared to infants without any sepsis.

**Conclusions:**

The incidence of neonatal SA sepsis has reduced over the last 20 years, suggesting potential effectiveness of the preventative interventions implemented. Endotracheal tube (ETT) colonisation and prolonged ventilation may be under-recognised as potential sources of SA infection. Our study suggests SA sepsis may negatively impact neurodevelopmental outcomes.

## Introduction

Neonatal *Staphylococcus aureus* (SA) sepsis can be defined as blood culture(s) positive with SA in an unwell baby during the first month of life [1]. Clinical signs of SA sepsis are often non-specific including temperature instability, bradycardia, oxygen desaturation, poor feeding, and irritability [2]. Late-onset sepsis (LOS) is defined by the Australian and New Zealand Neonatal Network as occurring beyond 48 h of life, while early-onset sepsis (EOS) presents within the first 48 h [3]. Approximately 70% of neonatal LOS is caused by Gram-positive bacteria, Coagulase-negative staphylococci being responsible for approximately two-thirds of these, followed by SA as the second most frequent causative organism [1]. SA sepsis can cause serious and life-threatening complications including pneumonia, osteomyelitis, septic arthritis, and endocarditis [1, 4]. LOS with any organism substantially increases the risk of neurodevelopmental impairment [5].

SA sepsis is a recognised cause of hospital-acquired infections and monitored in Western Australia (WA) via mandatory reporting to the state Healthcare Infection Surveillance program [6]. There is currently no routine screening program for SA in WA NICUs outside of an outbreak [7]. Prolonged hospitalisation, long-term respiratory support, and invasive procedures such as central lines and endotracheal tubes (ETT) are risk factors for SA sepsis in neonates [1]. Low gestational age (GA) and birth weight (BW) are further established independent risk factors for LOS [1, 8].

No consensus exists on the best empirical regimen of antibiotics for neonatal sepsis and the choice should be based on local microbiology and epidemiology data [1]. Local protocol in WA suggests empirical Vancomycin and Gentamicin for LOS and Penicillin and Gentamicin for EOS until blood cultures and susceptibility testing are reported [7].

The aim of this study was to determine the clinical characteristics of neonatal SA sepsis in WA over the past 20 years, examine presence of risk factors for LOS within this cohort, and compare selected short- and long-term outcomes to control infants without any sepsis.

## Methods

### Location

The only tertiary NICU in WA.

### Design

Retrospective cohort study with embedded case-control analysis for specific short- and long-term outcomes. The study was approved by the institutional research ethics committee (#33899).

### Inclusion criteria

All neonates cared for in the only tertiary NICU for Western Australia with proven SA sepsis (defined as: blood cultures positive for SA in the presence of clinical signs and antibiotic treatment duration ≥5 days) between 2001 and 2020 were included.

### Exclusion criteria

Nil.

### Data collection

Infants with proven SA sepsis between 2001 and 2020 were identified in routine electronic databases and demographic information extracted, including GA, BW, sex, and clinical outcomes. Clinical signs, complications, invasive line duration and antibiotic selection, and duration of administration were extracted from neonatal and maternal medical records. Further information regarding microbiological results were obtained from the electronic pathology database, including sensitivities of SA blood culture isolates, and testing for bacterial colonisation. Neurodevelopmental outcomes at 2 to 5 years of age were extracted from the developmental follow-up database.

### Outcomes

Infants with SA sepsis were matched 1:2 with control infants without any sepsis. Infants were matched by gestational age at birth, sex, and year of birth to reduce bias and account for changes in general practice over time. Non-parametric continuous data were summarised using median, interquartile range (IQR) and range (R), and categorical data summarised with frequency distributions. Univariate continuous outcomes were analysed using linear regression with robust standard errors and categorical outcomes were analysed using simple conditional logistic regression to account for matched clusters. BW and GA were transformed to the natural logarithm to correct for non-normality in analysis. Duration of total parenteral nutrition (TPN), ventilatory outcomes and length of hospital admission were analysed using cox proportional hazards regression using the ‘shared-frailty’ grouping function to account for the matching. Deaths were censored in analysis. Models were adjusted for covariates determined to influence outcomes including GA, BW z-score, year of birth and inborn status. Estimates were presented using hazard ratios and their 95% confidence intervals (CI). Neurodevelopmental outcomes were analysed using independent group tests due to the smaller sample size for these measures. Bayley composite scores (<85 and <70) were compared using Chi-square or Fisher exact tests. Disability was defined as a cognitive composite score <70 or Full-Scale Intelligence Quotient (FSIQ) < 70 (<2 SD below the mean). Continuous scaled scores in each domain were compared univariately using t-tests and further analysed using multiple linear regression with adjustments for gestation at birth, BW z-score and corrected age at assessment. Binary composite score outcomes were not adjusted in modelling as event rates were low and numbers were insufficient to analyse further. Stata version 16 (StataCorp, Texas) statistical software was used for data analysis.

## Results

### Incidence and demographics

Over the 20-year study period approximately 4% of blood culture-positive infections in neonates were due to SA sepsis with an overall incidence 0.10/1000 live births (62/614207). Incidence of SA sepsis was significantly higher among very preterm infants (<32 weeks; 6.87/1000 live births) compared to 0.03/1000 for infants ≥32 weeks [9]. The incidence of SA sepsis fell from 0.13/1000 live births during 2001–2010 to 0.07/1000 live births from 2011–2020. MSSA accounted for 46 (74%) cases while 16 (26%) were caused by MRSA.

Ninety percent (56/62) of SA sepsis episodes occurred in preterm infants (<37 weeks GA) and most affected infants born very preterm (<32 weeks GA; 76%, 47/62). The median GA at birth of affected infants was 28 weeks (R 23–41; IQR 25–31). Most SA sepsis, 87% (54/62) occurred in infants of low BW (<2500 g), with 73% (45/62) in infants of very low BW (<1500 g) and 52% (32/62) in infants of extremely low BW (<1000 g). The median BW was 963 g (range 424–4080 g; IQR 686–1542).

Neonates of Aboriginal or Torres Strait Islander descent made up 13% (8/62) of our cohort. In indigenous infants, 88% (7/8) of SA sepsis cases were caused by MSSA and 12% (1/8) by MRSA. All presented as LOS. Two (25%) died in hospital.

After matching uninfected control infants to SA sepsis cases (2:1) there was a total study cohort of 186 infants. Demographic characteristics were similar between the groups as seen in Table 1.

**Table 1:** Neonatal characteristics of SA sepsis and infants without sepsis.

Variable	SA Sepsis *N* = 62	Infants without sepsis *N* = 124	*p*-value
Gestational age (w)	28 (25–31; 23–41)	28 (25–30; 23–41)	0.78
Birth weight (g)	963 (686–1542;424–4080)	1070 (776–1601;445–4700)	0.10
Birth weight z-score	0.00 (−0.7–0.7;−2.74–2.79)	0.31 (−0.43–0.99;−2.89–2.62)	0.15
Male	32 (52%)	64 (52%)	1.00
Inborn	55 (89%)	113 (91%)	0.19
Died before discharge	4 (7%)	6 (6%)	0.64

Data represents median, interquartile range and range or number (%), as appropriate.

*SA* Staphylococcus aureus.

### Early-onset sepsis

EOS accounted for 9.7% (6/62) of SA sepsis with an incidence of 0.01 per 1000 live births. All were MSSA cases. The median GA was 36 weeks (range, 26–41; IQR, 33–40) with a median BW of 2985 g (range, 780–4080; IQR 1935–3990). Delivery was by C-section for 5 infants and by spontaneous vaginal delivery for 1 infant. Five cases of EOS had maternal risk factors including fever (*N* = 2), chorioamnionitis (*N* = 2), meconium-stained liquor (*N* = 1), and prolonged ruptured membranes for 72 h (*N* = 1), and confirmed maternal colonisation with SA on vaginal swab and/or placental screening was present for 4 (67%) cases. No deaths were recorded.

### Late-onset sepsis

**Fig. 1:**
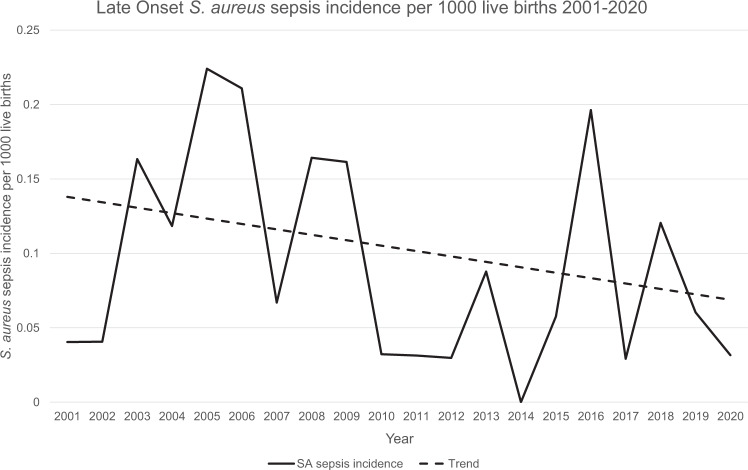
Temporal incidence of late-onset SA sepsis per 1000 live births with trend line.

The majority of SA cases were classified as LOS (56/62, 90%) with a median age at onset of 14 days (range, 4–90; IQR, 10–19). The incidence of LOS was 0.09 per 1000 live births with the temporal incidence trending down, shown in Fig. 1. MSSA accounted for 40 (71%) cases with 16 (29%) caused by MRSA. The incidence of LOS for GA < 32 weeks was 6.7/1000 live births compared to 0.02/1000 live births for GA ≥ 32 weeks. The median GA was 27 weeks (range, 23–41; IQR 25–30) with a median BW of 928 g (range, 424–3645; IQR, 675–1404).

### Associated factors with *S. aureus* sepsis

A summary of previously identified risk factors associated with neonatal SA sepsis within this cohort can be seen in Table 2, stratified by onset of sepsis, GA, and organism resistance. Overall, most infants were preterm (90%) with low GA and BW. 40 (65%) infants were delivered via caesarean section and maternal colonisation with SA confirmed via microbiology results was seen in 8 cases. A majority of infants received TPN at the time of sepsis (37/62, 60%) and 81% (50/62) had either a central line (16/62, 26%) or peripheral intravenous (IV) cannula (34/62, 55%) in situ. 19/62 (31%) of infants were intubated and received mechanical ventilation at the time of sepsis. Infants born at GA < 32 weeks were more likely to have had a peripheral IV cannula in situ or to require administration of TPN or invasive ventilation at the time of SA sepsis than more mature infants.

**Table 2:** Associated factors for neonatal SA sepsis present at the time of positive blood culture.

Associated Risk Factors	EOS (*N* = 6) (%)	LOS (*N* = 56) (%)	GA < 32 weeks (*N* = 47) (%)	GA ≥ 32 weeks (*N* = 15) (%)	MSSA (*N* = 46) (%)	MRSA (*N* = 16) (%)	Total, *N* (% of total cohort, *N* = 62)
Preterm birth < 37 weeks	3 (50%)	53 (95%)			40 (87%)	16 (100%)	56 (90%)
Very preterm (28–32 weeks)	0 (0%)	15 (27%)			9 (20%)	6 (38%)	15 (24%)
Extremely preterm (<28 weeks)	1 (1%)	31 (55%)			23 (50%)	9 (56%)	32 (52%)
Very low birth weight (<1500 g)	1 (1%)	44 (79%)	44 (94%)	1 (7%)	31 (67%)	14 (88%)	45 (73%)
Proven colonised with SA prior to blood culture^a^	2 (2%)	15 (27%)	16 (34%)	1 (7%)	12 (26%)	5 (31%)	17 (27%)
Central lines in situ^b^	2 (33%)	14 (25%)	12 (26%)	4 (27%)	12 (26%)	4 (25%)	16 (26%)
- UAC	1	1	2	0	1	1	2
- UVC	1	3	2	2	3	1	4
- PICC	0	10	8	2	8	2	10
IV cannula in situ^b^	1 (1%)	33 (59%)	29 (62%)	5 (33%)	23 (50%)	11 (69%)	34 (55%)
ETT in situ^b^	0 (0%)	19 (34%)	19 (40%)	0 (0%)	14 (30%)	5 (31%)	19 (31%)
TPN in situ^b^	0 (0%)	37 (66%)	33 (70%)	4 (27%)	25 (54%)	12 (75%)	37 (60%)
Surgical procedures prior to sepsis	0 (0%)	2 (4%)	1 (2%)	1 (7%)	1 (2%)	1 (6%)	2 (3%)

^a^positive blood culture for SA

^b^at time of positive SA on blood culture

*EOS* early onset sepsis, *ETT* endotracheal tube, *GA* gestational age, *LOS* late onset sepsis (>48 h), *MRSA* methicillin resistant Staphylococcus aureus, *MSSA* methicillin susceptible Staphylococcus aureus, *N* number of cases (%), *PICC* peripherally inserted central catheter, *SA* Staphylococcus aureus, *TPN* total parenteral nutrition, *UAC* umbilical artery catheter, *UVC* umbilical vein catheter

The median duration of umbilical artery catheters at the time of positive blood culture was 5.5 days (range 3–8) and 4 days (range 2–8) for umbilical venous catheters. The median duration of peripherally inserted central catheters (PICC) before positive blood cultures was 10 days (range, 4–32 days) and for peripheral IV cannulas was 2 days (range, 0–6). Three of the infants with a PICC line in situ had documented surrounding skin inflammation at time of sepsis.

### Colonisation with *S. aureus*

There is no routine universal screening specifically for SA in Western Australia, unless there is an outbreak or known maternal MRSA colonisation, and as a result many cases of SA colonisation will remain unknown. Current general screening practice (not specifically for SA) includes gastric aspirate and ear swab cultures upon admission the NICU plus ETT aspirate cultures weekly for intubated infants. Otherwise, testing is as clinically indicated for example if discharges or inflammations are detected. Of the 62 SA sepsis cases, in the majority the first detection of SA was on blood culture (45/62, 72.6%). However, in 8 infants (12.9%) SA colonisation was first noted on ETT aspirates, routinely collected weekly to monitor microbial colonisation of intubated patients. Other sites of colonisation detected prior to positive blood cultures included ear swab (4/62, 6.5%), gastric aspirate (2/62, 3.2%), skin (3/62, 4.8%) and eyes (5/62, 8.1%). Testing was by clinical indication, thus not all infants had results for every site. Of those colonised with SA prior to sepsis, the median time from first SA detection to positive blood culture was 7 days (range 1–25 days).

### Focal signs and dissemination

The first documented clinical presentations for most infants (49/62, 79%) were non-specific, including features such as bradycardia, oxygen desaturations, temperature instability, poor feeding, and irritability. Six infants had skin or soft tissue inflammation at presentation, three of which were documented to be around invasive line sites.

Focal signs and/or complications occurred in 23 infants (37.1%) of which 18 cases were MSSA-related, with 14 skin, soft tissue, joints and/or bone infections recorded. Conjunctivitis (*n* = 9) and pneumonia (*n* = 8) were also common.

### Management

In our unit, blood culture-positive infections are treated with antibiotics for a minimum of five days but if infection persists or is suspected at other sites, duration of treatment is extended based on multidisciplinary assessment. Empiric antibiotic therapy included both Vancomycin and Gentamicin in 87.5% (49/56) of infants with LOS, as recommended by hospital policy. For infants with EOS 4 out of 6 received a Penicillin (Penicillin, Benzylpenicillin, or Flucloxacillin) and Gentamicin, with the remaining two infants either receiving one or the other of these two antibiotics. In cases of MSSA, including both EOS and LOS, 38 infants (73.9%) were changed to targeted monotherapy with Flucloxacillin. All MRSA cases had Vancomycin included in the empirical first-line therapy. The overall median duration of total antibiotic therapy given was 9 days (range 1–53 days).

### Neonatal outcomes

Neonatal outcomes with available data were compared to control infants (Table 3). The median duration of ventilation and on TPN was higher among infants with SA sepsis compared to uninfected infants (ventilation: 31 vs 18 days, aHR 2.22, 95% CI 1.54–3.23, *p* < 0.001 and TPN days: 14 vs 7 days, aHR 2.50, 95% CI 1.75–3.57, *p* < 0.001); these were adjusted for GA, BW z-score, inborn status, and year of birth in order to reduce bias and account for changed in general practice over time. Duration of oxygen supplementation and length of hospital admission were similar between groups.

**Table 3:** Descriptive summaries (median, interquartile range (IQR), and range (R)) and unadjusted and adjusted hazard ratios (HR)^c^ and 95% confidence intervals (CI) for durations of TPN, respiratory outcomes, and length of nursery stay.

Variable	Median (IQR, R)	Unadjusted HR 95% CI	*p*-value	Adjusted HR^a^ 95% CI	*p*-value
Ventilation duration (days)					
Infants without sepsis	18 (1–53; 0–105)	1.00		1.00	
SA Sepsis	31 (6–64; 0–166)	2.33 (1.59–3.45)	**<0.001**	2.22 (1.54–3.23)	**<0.001**
TPN duration (d)					
Infants without sepsis	7 (0–13; 0–67)	1.00		1.00	
SA Sepsis	14 (6–24; 0–165)	2.08 (1.49–2.94)	**<0.001**	2.50 (1.75–3.57)	**<0.001**
Oxygen duration (days)					
Infants without sepsis	4 (0–53; 0–146)	1.00		1.00	
SA Sepsis	9 (0.3–77; 0–200)	1.18 (0.29–4.76)	0.822	1.04 (0.26–4.17)	0.954
Length of nursery stay (days)					
Infants without sepsis	61 (18–94; 0–146)	1.00		1.00	
SA Sepsis	76 (39–113; 2–200)	1.10 (0.28–4.35)	0.888	0.99 (0.25–4.00)	0.995

^a^Adjusted for gestational age at birth, birth weight z-score, inborn, and year of birth.

*CI* confidence interval, *HR* hazard ratio, *IQR* interquartile range, *R* range, *SA* Staphylococcus aureus, *TPN* total parenteral nutrition

### Neonatal mortality

Four infants (6.2%) with SA sepsis died, three of which were clinically attributed to the infection. Two infants developed pneumonia and one developed bacterial endocarditis, osteomyelitis, and cellulitis. The gestational ages at birth of the three sepsis-related deaths were 23, 24, and 28 weeks. All were cases of LOS caused by MSSA occurring on day 9 of life, with a median time from sepsis presentation to death of 1 day (IQR 1–10). All were receiving invasive ventilation and TPN at the time of SA sepsis; two had central lines present and none had surgery. A fourth infant born at 23 weeks had sepsis at 76 days and recovered but died at 165 days due to severe bronchopulmonary dysplasia.

### Neurodevelopmental outcomes

Bayley Scales of Infant Development scores at 24 months corrected age were available for a subgroup of children who qualified for routine developmental assessment. There were 19 SA sepsis infants and 63 uninfected infants who had either a Bayley assessment at 24 months corrected age and/or a Wechsler Preschool and Primary Scale of Intelligence FSIQ assessment score at 5 years as seen in Table 4 with domain scores in Supplementary Table 1. The most recent assessment was used. There were 2/19 (10.5%) sepsis infants who had an overall disability score <70 compared with 3/63 (4.8%) without sepsis (*p* = 0.585). Cognitive scores <85 were more common among infants with proven LOS (26.7% vs 3.7%, *p* = 0.018).

**Table 4:** Neurodevelopmental outcomes.

Variable	Sepsis *N* = 19	No sepsis *N* = 63	*p*-value
Gestational age at birth (weeks)	26.1 (range: 24–30)	26.1 (range: 23.4–32.3)	0.834
Corrected age at assessment (months)	23.9 (range: 22.4–25.1)	24.0 (range: 22.0–36.0)	0.193
Bayley Assessment			
*Composite scores*	*N* = 15	*N* = 54	
Cognitive			
<85	4 (26.7%)	2 (3.7%)	0.018
<70	2 (13.3%)	1 (1.9%)	0.117
Language			
<85	5 (35.7%)	19 (36.5%)	0.955
<70	2 (14.3%)	8 (15.4%)	1.000
Motor			
<85	3 (20%)	6 (11.5%)	0.671
<70	2 (13.3%)	2 (3.8%)	0.214
FSIQ			
<70	0/7 (-)	2/26 (7.7%)	1.000
Overall disability^a^	2/19 (10.5%)	3/63 (4.8%)	0.585

^a^Overall disability included cognitive composite score <70 or Full-Scale Intelligence Quotient (FSIQ) < 70.

Data represent median and range or number (%), as appropriate.

## Discussion

SA sepsis is an ongoing cause of serious neonatal morbidity and mortality [1, 4, 10]. This retrospective cohort study aimed to characterise neonatal SA sepsis over the past 20 years in WA. The overall incidence of SA sepsis was 0.10 per 1000 live births. This was slightly higher than previously reported in Australia (0.08/1000 live births) between 1992 and 1999 by Isaacs et al [11]. In our cohort, the incidence was highest among infants born <32 weeks (6.87/1000 live births) and the majority of affected infants (90%) were born preterm and of very low birth weight (73%), consistent with previous studies [12, 13]. In our cohort, the ratio of MRSA/MSSA was 0.35, similar to that reported by Isaacs (0.34) for Australasia between 1995 and 1998 [11]. We did not observe higher mortality with MRSA strains as previously reported, with all deaths attributed to MSSA infections in our cohort [11].

The incidence of SA sepsis changed during the study period; from 0.13/1000 live births during 2001 to 2010 to 0.07/1000 live births from 2011 to 2020. We examined the temporal relationship of late-onset SA sepsis (the predominant presentation) and multiple preventive interventions targeting nosocomial infections like neonatal LOS during the study period. The observed reduction in incidence of SA sepsis can theoretically be attributed to a number of possible factors, however, the precise contribution of each was impossible to determine. Targeted preventive interventions included improvements in hand hygiene, reduction in the use of intravascular lines, earlier enteral feeding, probiotic supplementation for preterm infants, and introduction of care bundles and education around the care of central and peripheral invasive lines [14–19].

The National Hand Hygiene Initiative introduction coincided with a reduction in the incidence of SA sepsis from 2010 to 2013 seen in Fig. 1 [14, 15]. In a Swiss study, hand hygiene promotion was also linked with reduced healthcare-associated infections in neonates [16]. A meta-analysis by Rao et al also showed reduced incidence of LOS in preterm infants receiving probiotics [17]. Introduction of routine probiotics for preterm infants in 2011 may have also contributed to this reduction in SA sepsis within our cohort.

Central and peripheral lines are established risk factors for SA sepsis, along with TPN and prolonged ventilation [1, 4, 20]. We found that the site most frequently colonised with SA prior to sepsis was the endotracheal tube and infants with SA sepsis had significantly longer median duration of ventilatory support than infants without sepsis (*p* < 0.001). This suggests presence of an endotracheal tube and prolonged invasive ventilation may be under-recognized as important risk factor or potential source of infection and supports findings from Antoine et al. demonstrating common bacterial colonisation of ventilation tubes in preterm infants and the association with sepsis [21]. Due to the routine weekly culture of ETT aspirates for infants intubated in the NICU and no routine swabs of other common sites of colonisation of SA such as the nasopharynx, this may contribute to why the ETT was the most frequently colonised site. TPN duration was also longer among neonates with sepsis than without sepsis (14 days vs 7 days respectively, *p* < 0.001). Our findings are in line with the concept that early introduction of enteral feeding lowers the risk of hospital-acquired infections [1, 22, 23].

Our analysis showed worse early childhood neurodevelopmental outcomes among neonates with SA sepsis, particularly in cognitive domains. These findings are consistent with previous reports of the negative impact of neonatal invasive bacterial infections on cerebral development [5, 24]. There is limited organism-specific literature investigating long-term outcomes after SA neonatal sepsis and further evaluation is needed.

Strengths of our study include the relatively large cohort examined for this condition over two decades during which different prevention strategies were implemented. Due to centralised tertiary neonatal intensive care in WA, our study resembles total population data. We also reported on neurodevelopmental outcomes for which there is limited evidence available.

Our study limitations include the retrospective nature and small number of infants included in long-term follow-up as this was a pragmatic retrospective study. Cautious interpretation of neurodevelopmental results is needed due to the small sample size. Additionally, not all mothers were tested for colonisation and not all sites of potential colonisation were routinely tested in all infants introducing bias toward tested sites. Those admitted to the NICU had gastric aspirates and ear swabs routinely, with an ETT aspirate taken if ventilated for prolonged periods. All other samples were taken based on clinical indication. As a result, neonatal colonisation prior to blood cultures and maternal colonisation with *S. aureus* may be under reported.

In conclusion, the incidence of particularly late onset SA sepsis has reduced over the last 20 years, suggesting potential effectiveness of the preventative interventions implemented. ETT colonisation and prolonged ventilation may be under recognised as potential sources of SA infection and our study suggests SA sepsis may negatively impact neurodevelopmental outcomes. There is an ongoing need for monitoring and investigation of particularly long-term effects of this serious infection and further potential preventative interventions.

## Acknowledgements

Maureen Hutchinson and Alan Joyce, Maternal and Child Health Management, Department of Health, WA for providing overall birth data.

## Supplementary information

Supplementary Table 1

## Data Availability

All data relevant to the study are included in the article or supplied as supplementary information.
