# Supplementary material for: Neonatal Staphylococcus Aureus Sepsis: a 20-year Western Australian experience
Source: J Perinatol. 2022 Jun 25;42(11):1440–5. doi: 10.1038/s41372-022-01440-3 (PMC9616716; doi:10.1038/s41372-022-01440-3)
Supplement: Supplementary file 1 — Supplementary Table 1 [file 41372_2022_1440_MOESM1_ESM.docx]

**Neonatal *Staphylococcus Aureus* Sepsis: a 20-year Western Australian Experience**

**SUPPLEMENTARY TABLE 1**

| Supplementary Table 1. Bayley scaled scores summarised with mean and standard deviation (sd) and adjusted mean effect estimates with 95% confidence intervals (CI). Adjustments were made for gestational age at birth, birthweight z-score and corrected age at time of assessment | | | | | |
| --- | --- | --- | --- | --- | --- |
|  | **Sepsis**  **N=15**  **Mean (sd)** | **No sepsis**  **N=54**  **Mean (sd)** | **p-value** | **Mean difference (95%CI)** | **p-value** |
| Bayley |  |  |  |  |  |
| *Scaled scores* | N=15 | N=54 |  |  |  |
| Cognitive | 8.6 (3.4) | 10.5 (2.9) | 0.032 | -1.8 (-3.7-0.1) | 0.064 |
| Language |  |  |  |  |  |
| Receptive | 8.1 (2.7) | 9.3 (4.3) | 0.355 | -0.7 (-3.3-1.9) | 0.587 |
| Expressive | 7.4 (2.8) | 8.5 (4.2) | 0.349 | -1.1 (-3.7-1.5) | 0.406 |
| Total language | 15.5 (5.3) | 17.8 (8.0) | 0.315 | -2.0 (-6.9-3.0) | 0.425 |
| Motor |  |  |  |  |  |
| Fine | 9.4 (3.1) | 11.1 (2.7) | 0.040 | -1.8 (-3.5-(-0.1)) | 0.038 |
| Gross | 7.8 (2.6) | 8.5 (2.4) | 0.323 | -0.4 (-2.0-1.1) | 0.564 |
| Total motor | 17.2 (5.1) | 19.6 (4.6) | 0.086 | -2.2 (-5.1-0.7) | 0.129 |
| Socio-emotional | 9.1 (2.7) | 10.7 (3.3) | 0.102 | -1.6 (-3.8-0.5) | 0.133 |

Bayley scaled scores in Table 5 were lower in survivors of SA sepsis both in the cognitive (p=0.038) and fine motor domains (p=0.04), which remained after adjustment for gestation at birth, BW z-score and corrected age at time of assessment.
